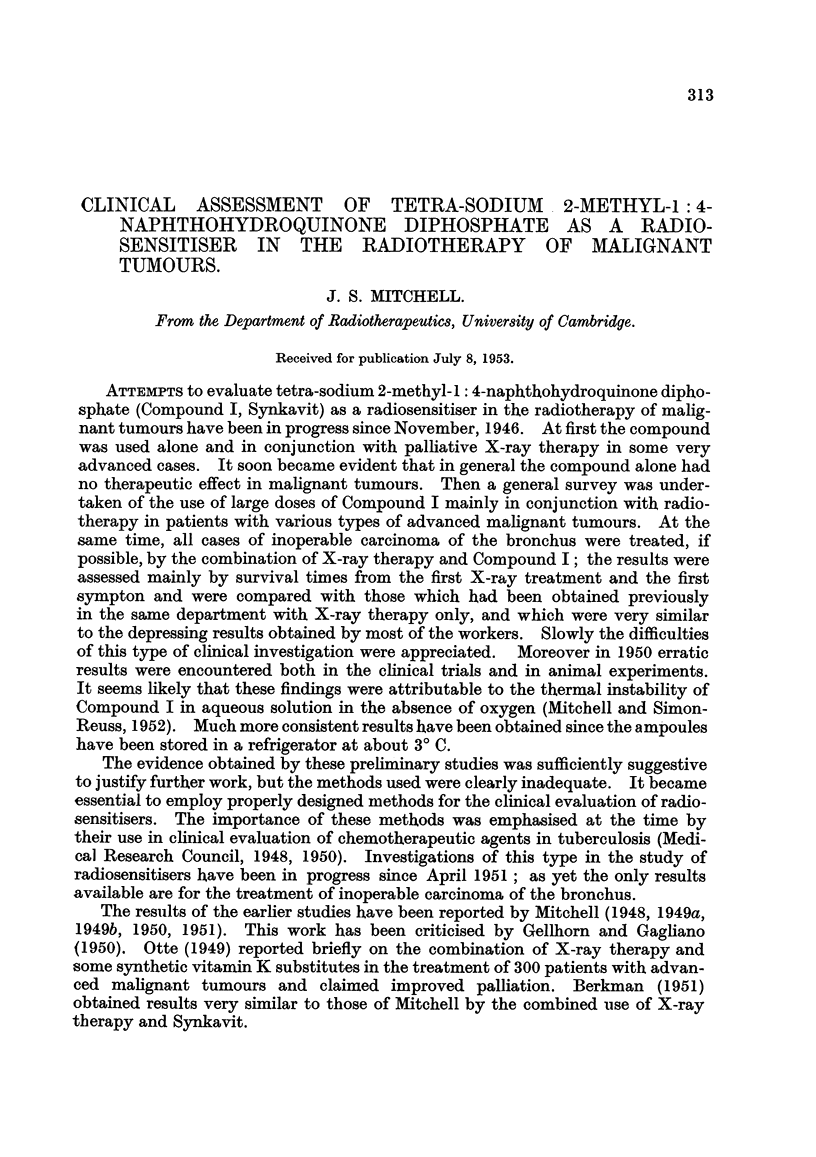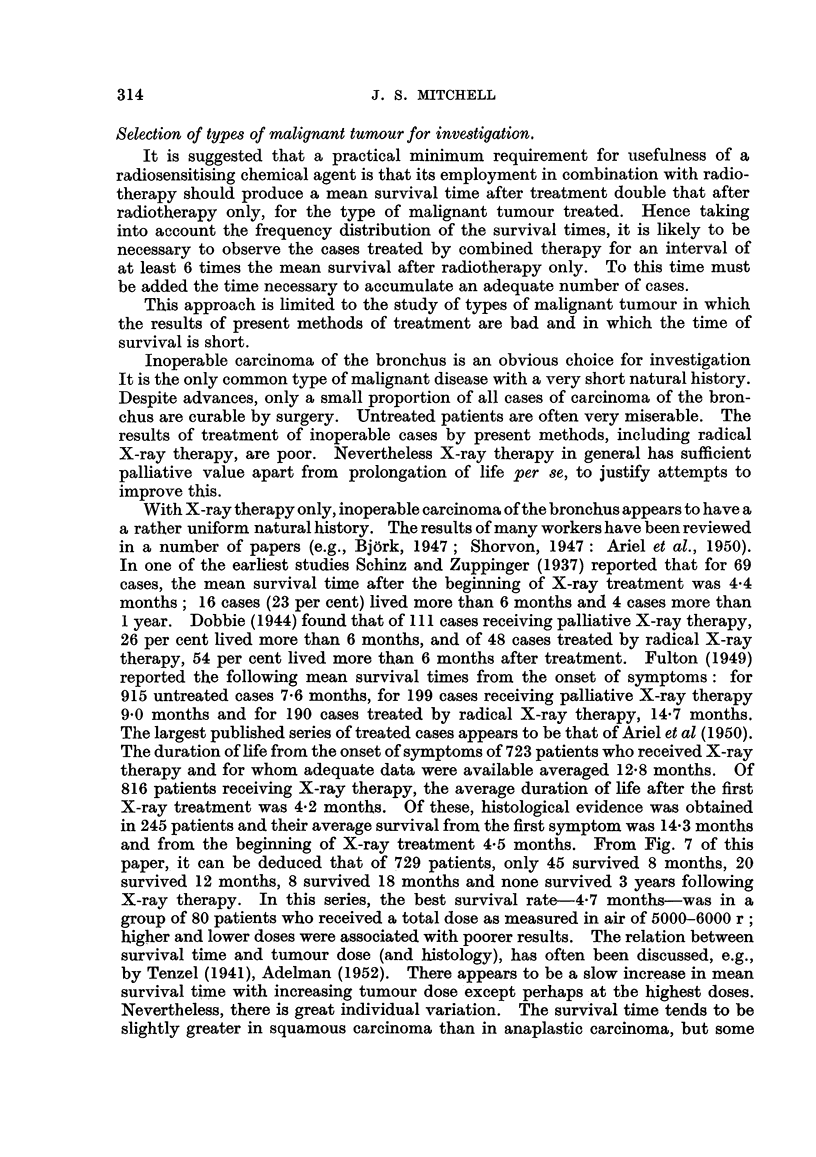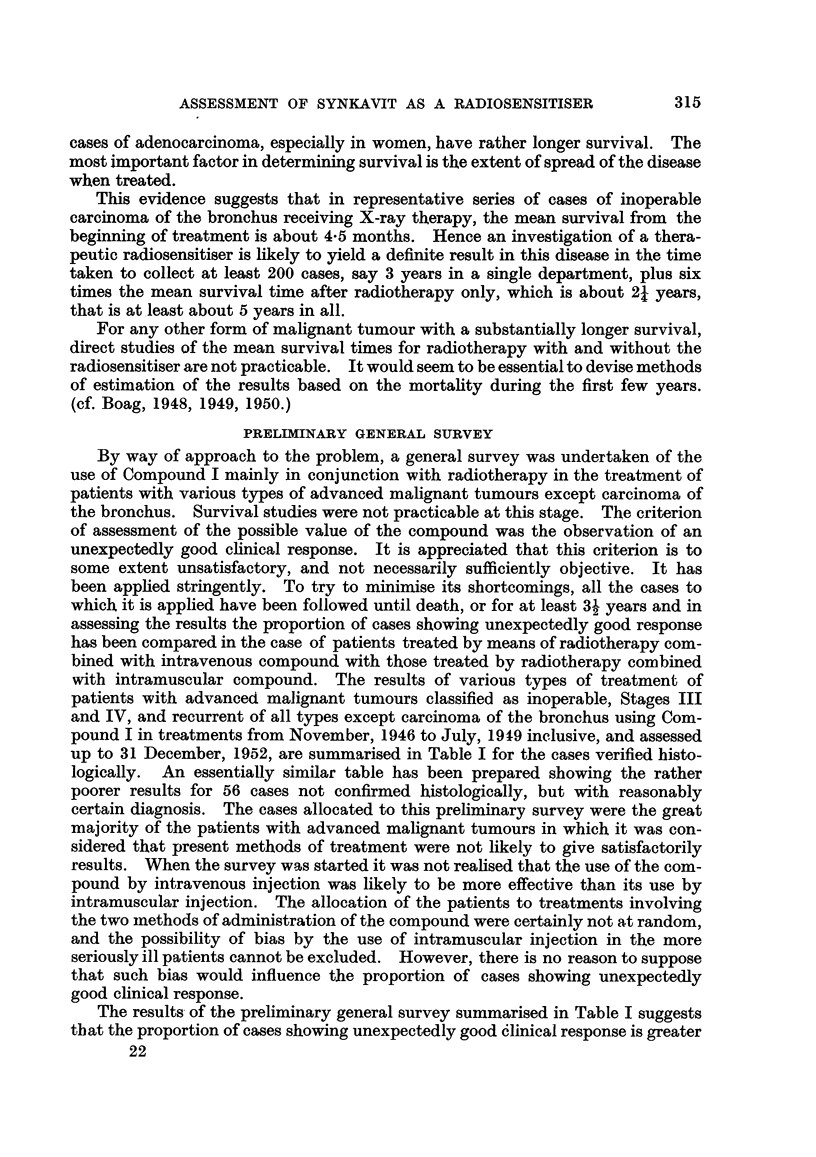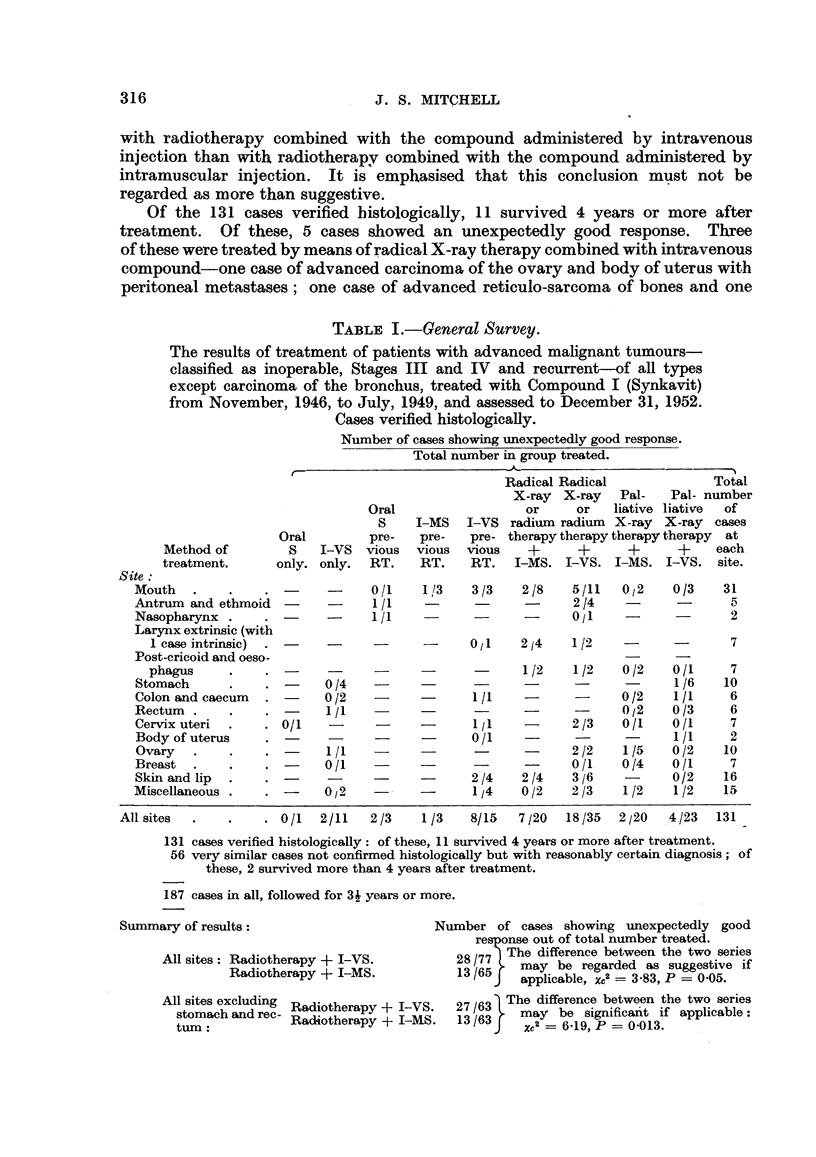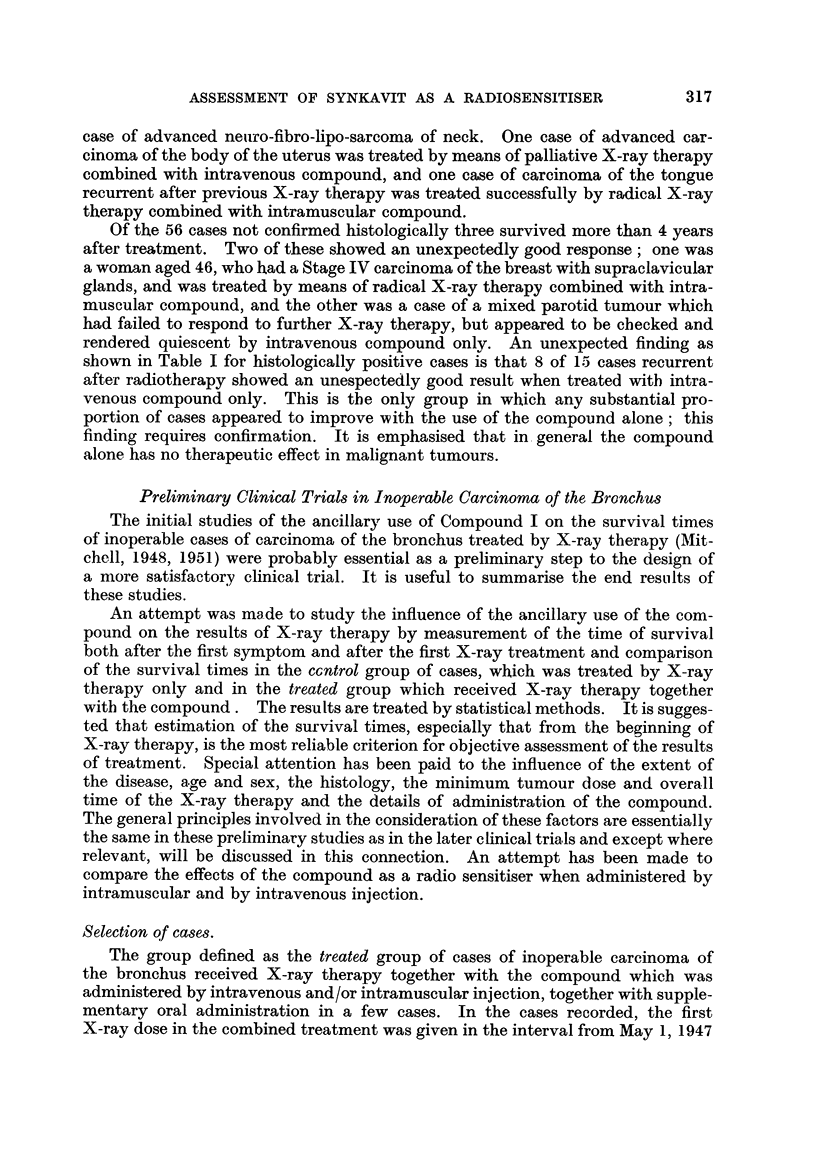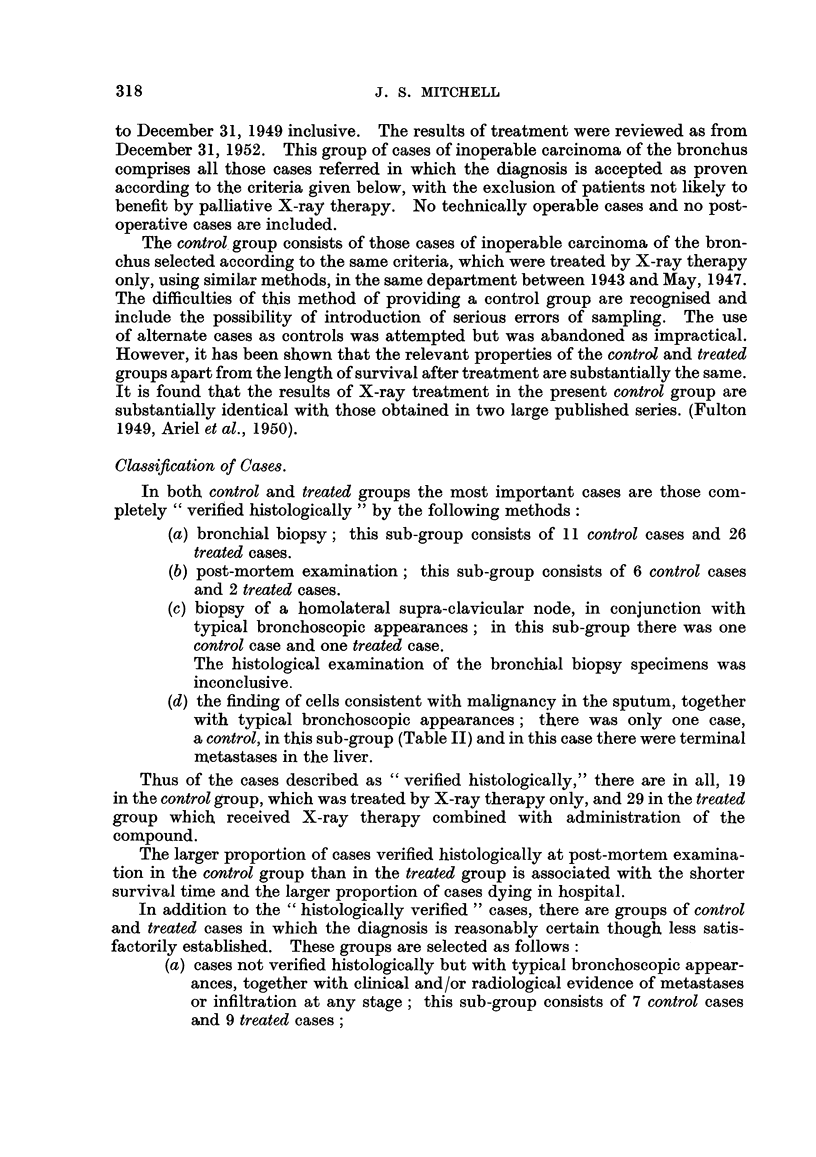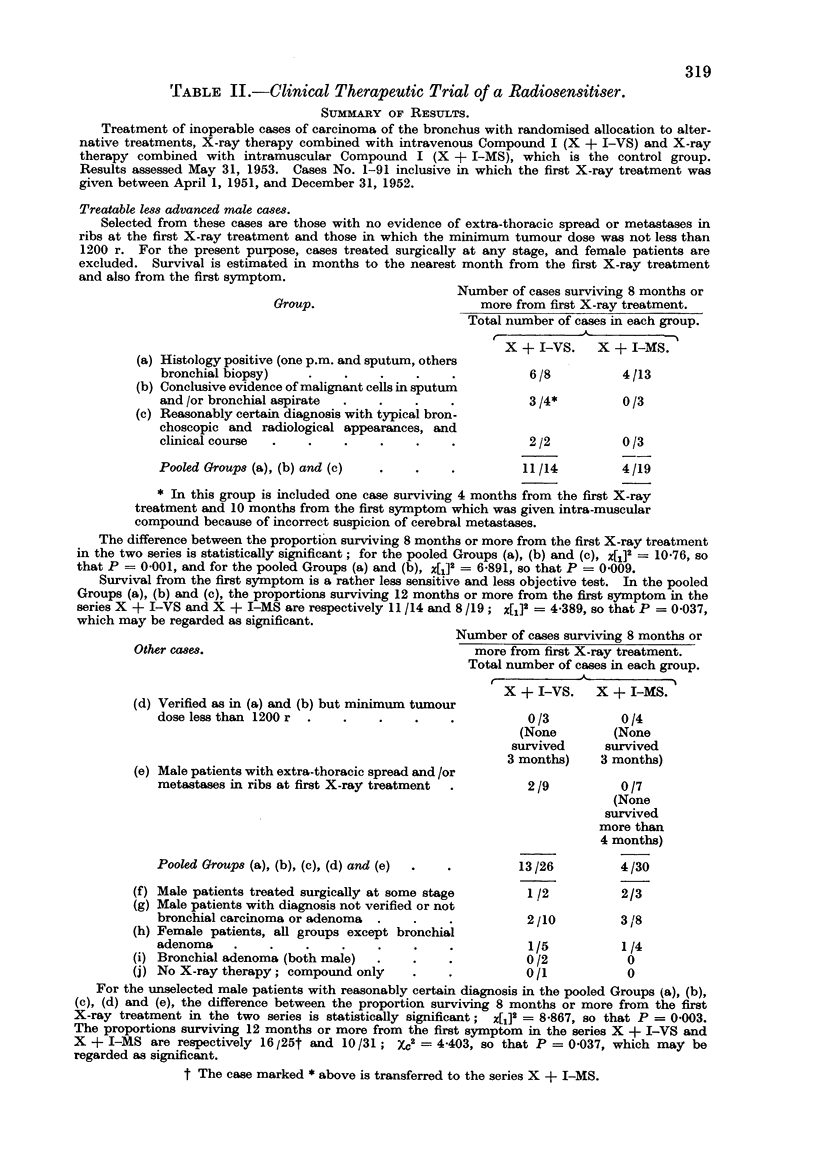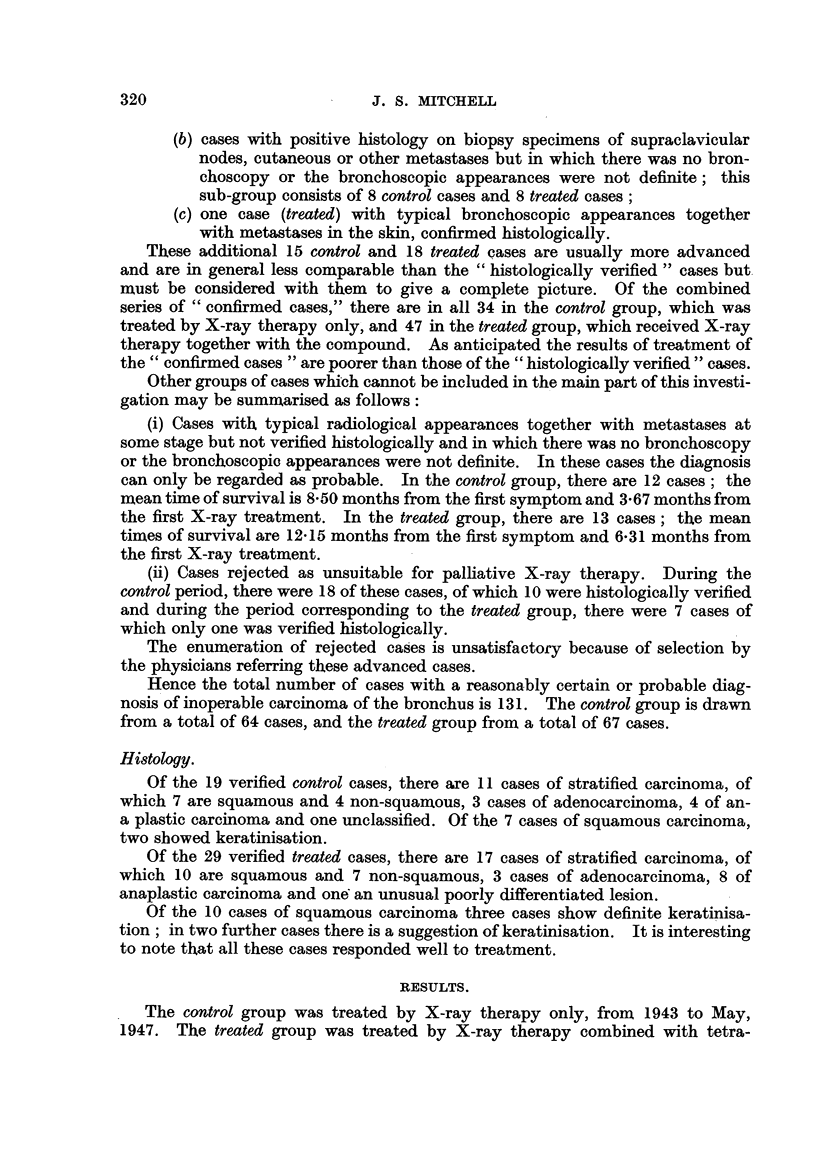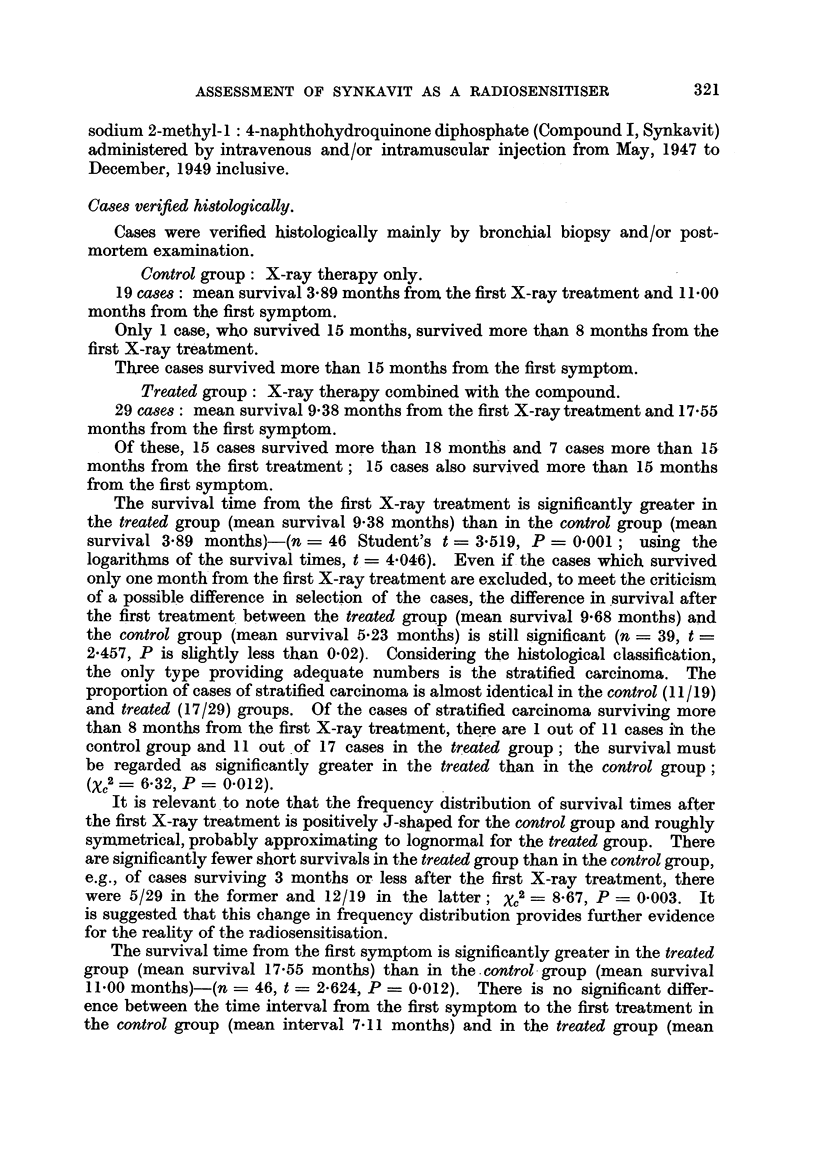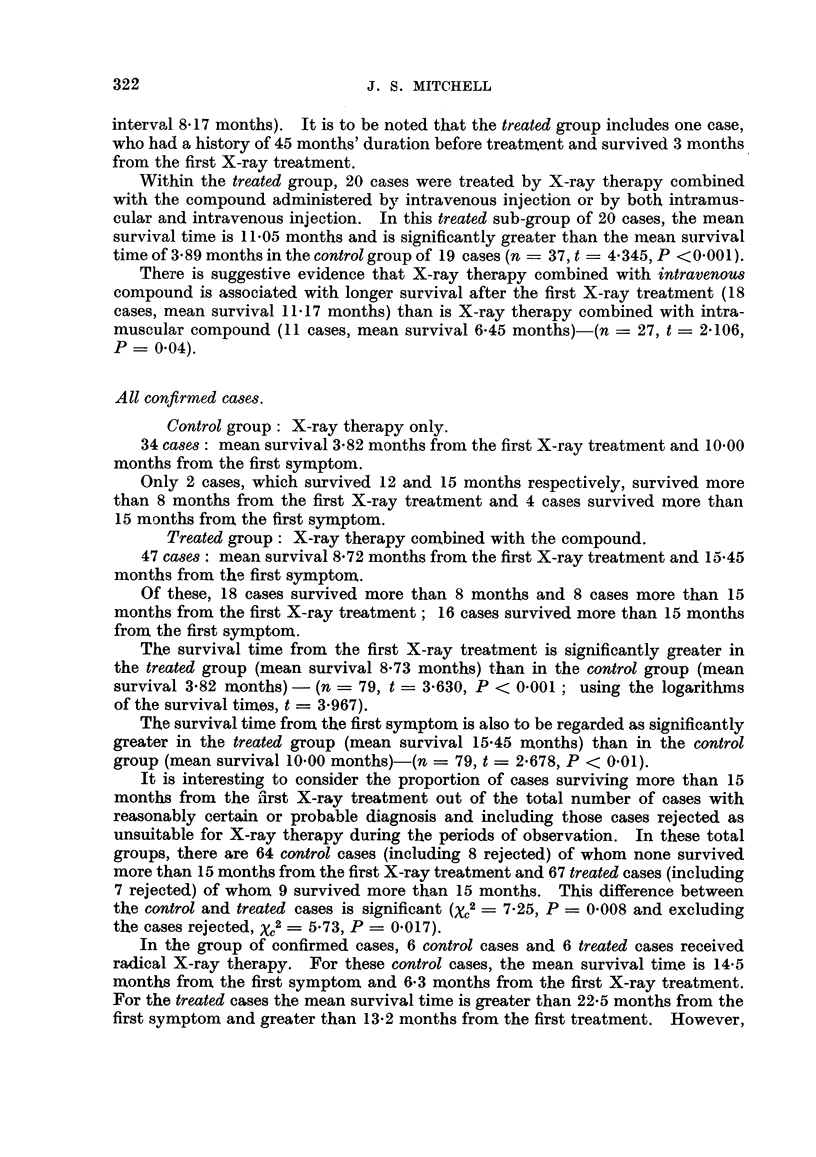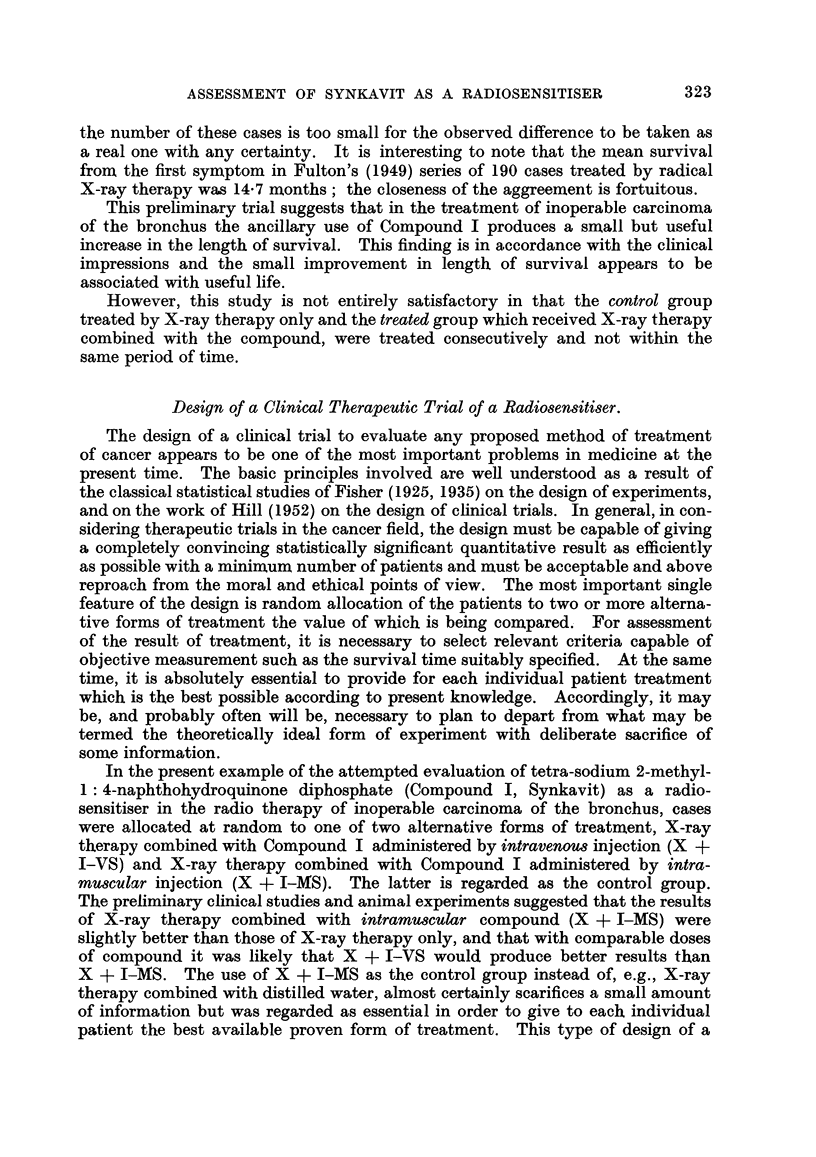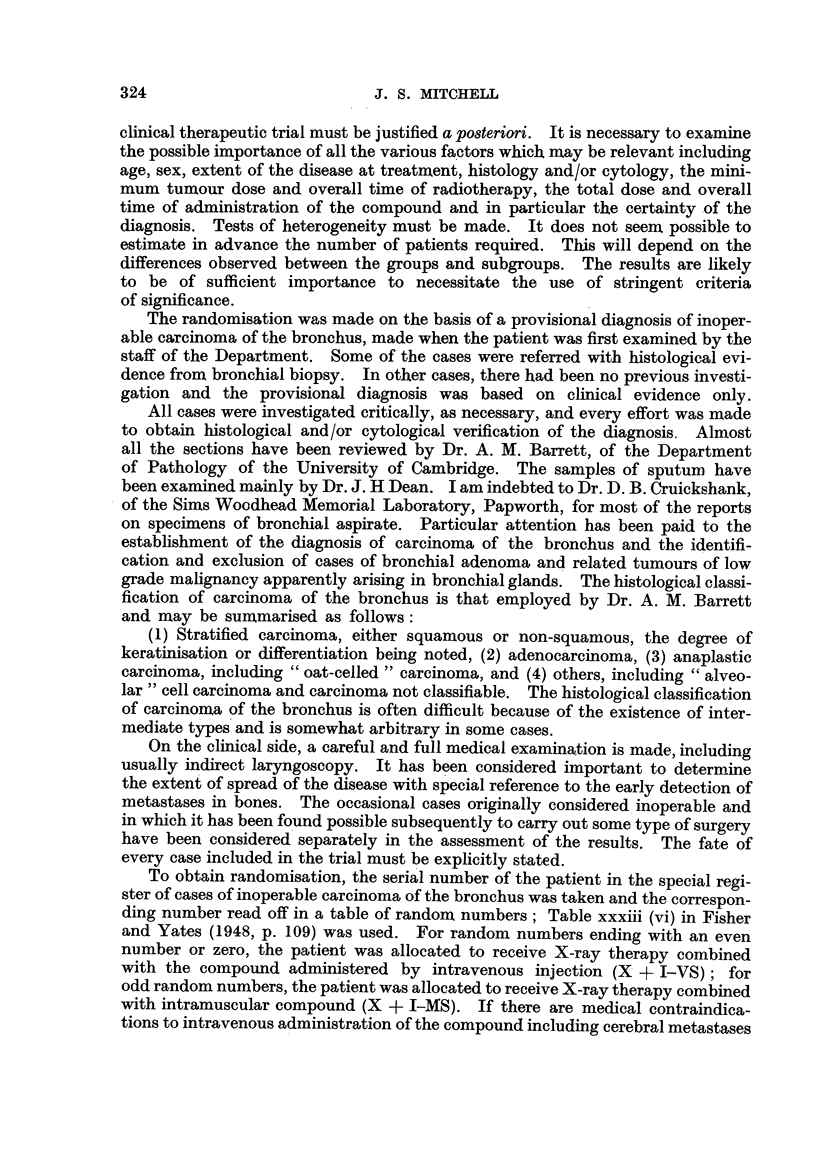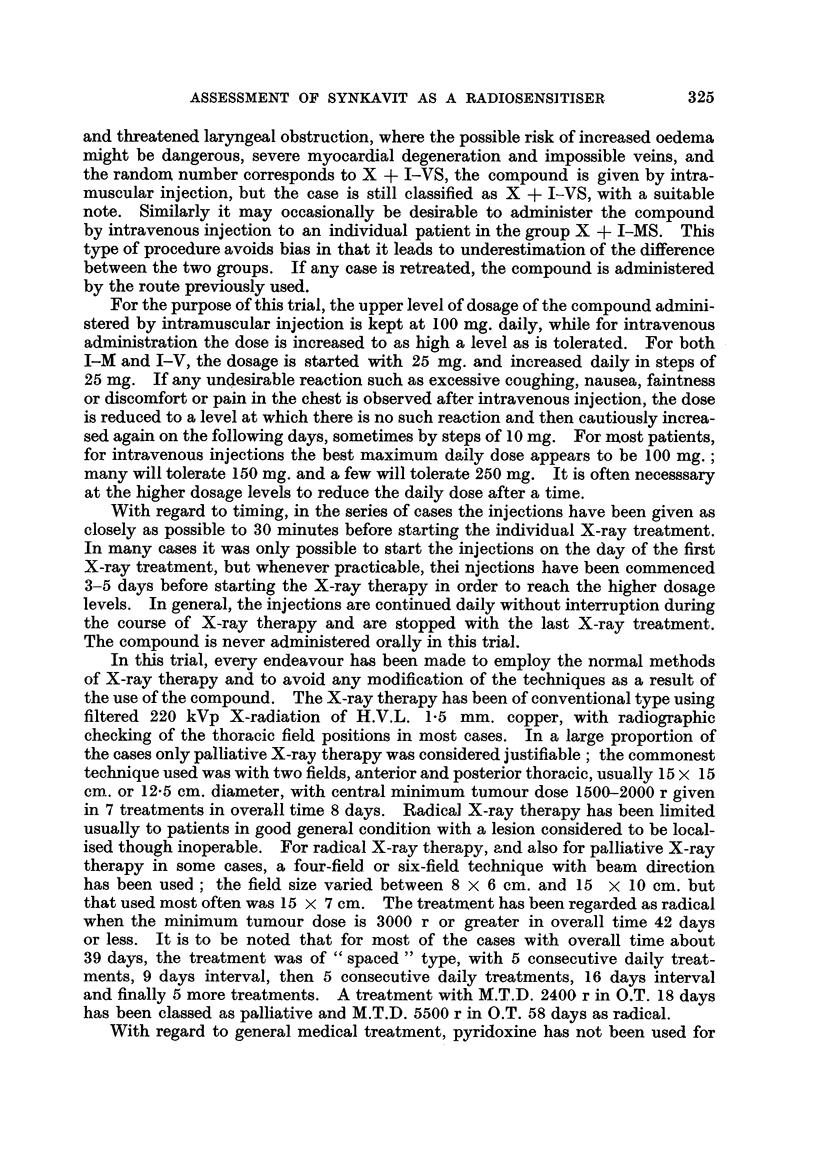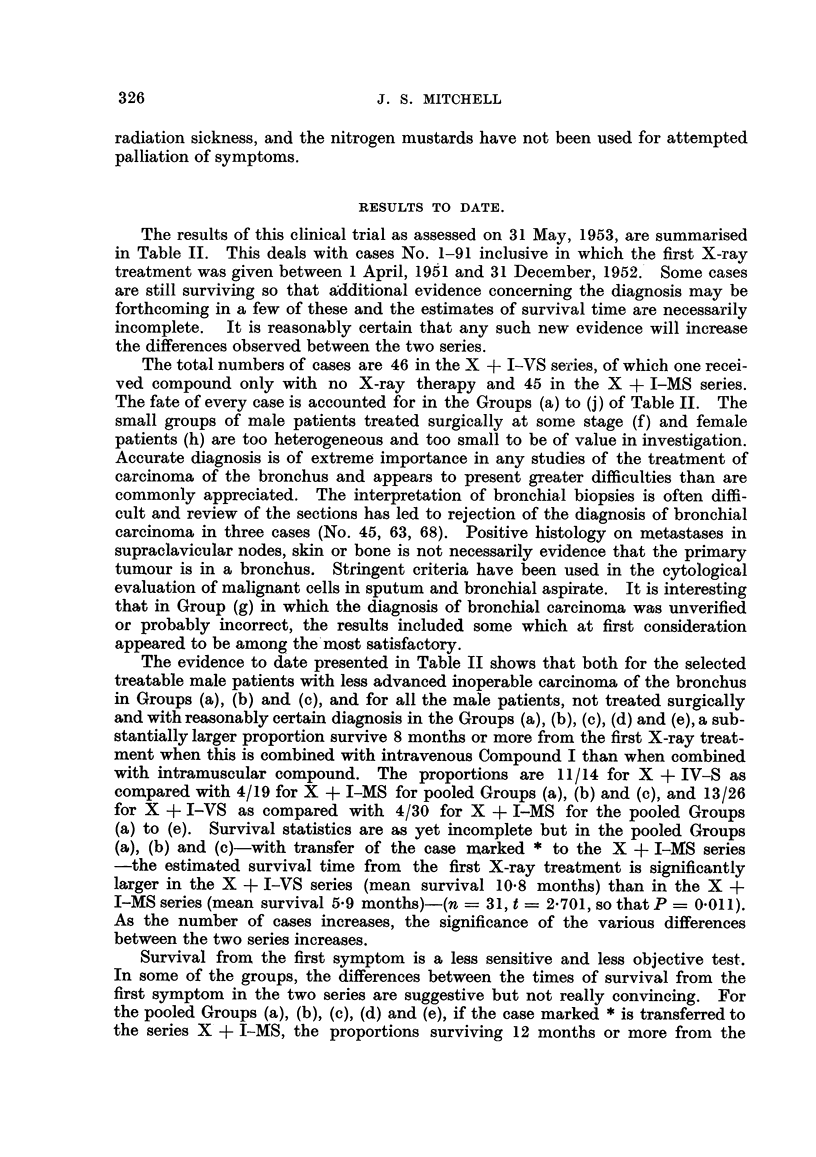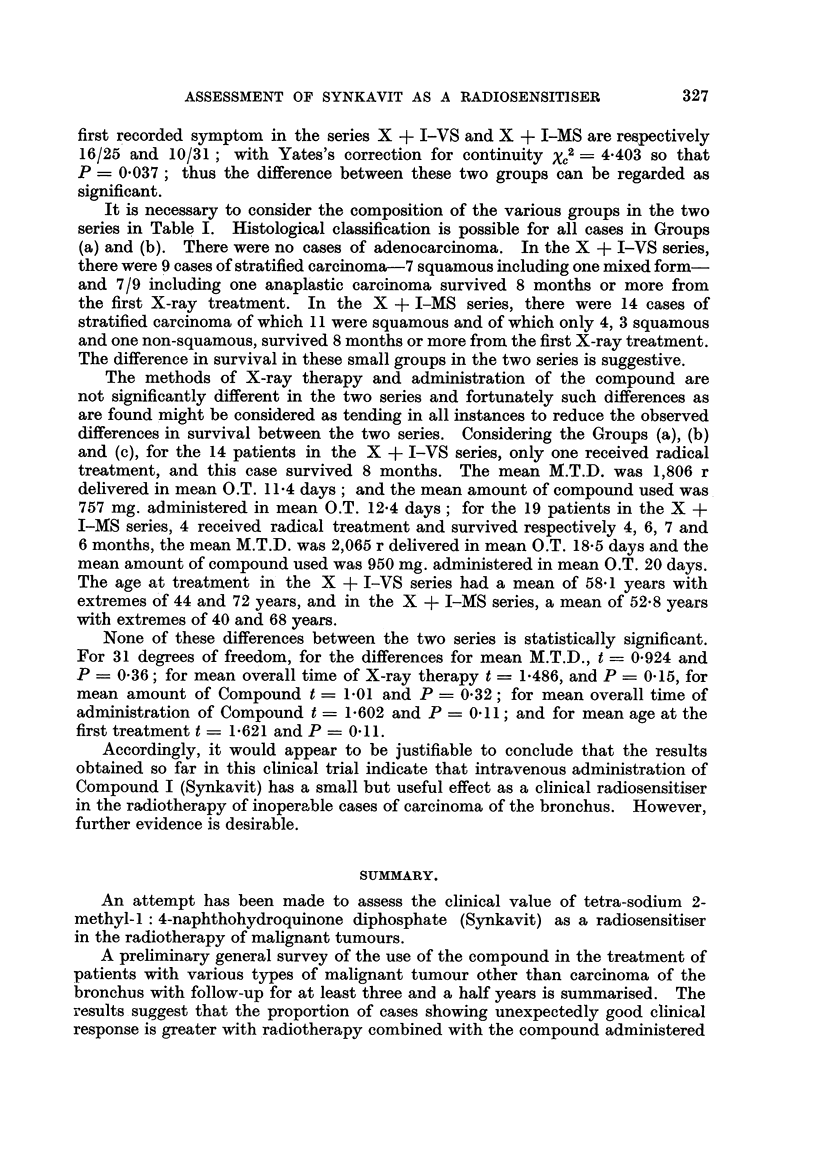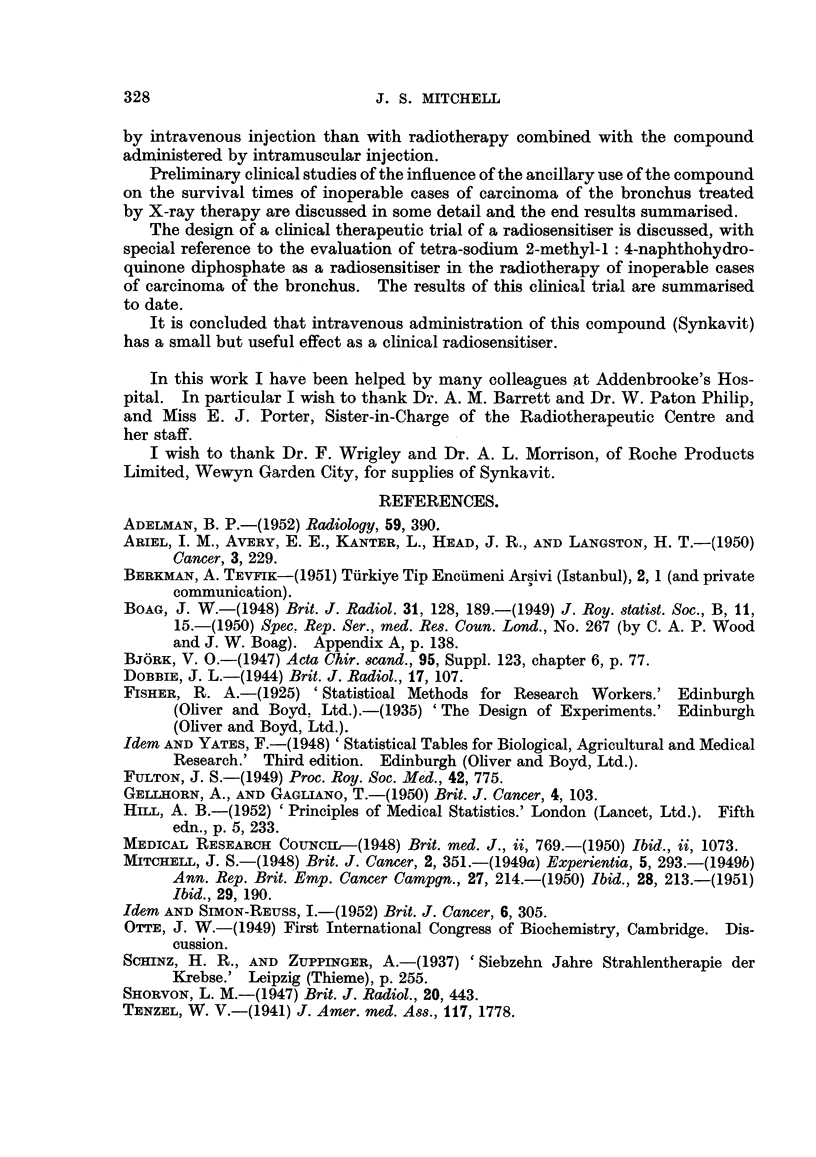# Clinical Assessment of Tetra-sodium 2-Methyl-1: 4-Naphthohydroquinone Diphosphate as a Radiosensitiser in the Radiotherapy of Malignant Tumours

**DOI:** 10.1038/bjc.1953.30

**Published:** 1953-09

**Authors:** J. S. Mitchell


### CLINICAL ASSESSMENT OF TETRA-SODIUM 2-METHYL-1 : 4-NAPHTHOHYDROQUINONE DIPHOSPHATE AS A RADIO-SENSITISER IN THE RADIOTHERAPY OF MALIGNANT TUMOURS.

J. S. MITCHELL.

*From the Department of Radiotherapeutics, University of Cambridge.*

Received for publication July 8, 1953.

ATTEMPTS to evaluate tetra-sodium 2-methyl-1 : 4-naphthohydroquinone diphosphate (Compound I, Synkavit) as a radiosensitiser in the radiotherapy of malignant tumours have been in progress since November, 1946. At first the compound was used alone and in conjunction with palliative X-ray therapy in some very advanced cases. It soon became evident that in general the compound alone had no therapeutic effect in malignant tumours. Then a general survey was undertaken of the use of large doses of Compound I mainly in conjunction with radiotherapy in patients with various types of advanced malignant tumours. At the same time, all cases of inoperable carcinoma of the bronchus were treated, if possible, by the combination of X-ray therapy and Compound I ; the results were assessed mainly by survival times from the first X-ray treatment and the first sympton and were compared with those which had been obtained previously in the same department with X-ray therapy only, and which were very similar to the depressing results obtained by most of the workers. Slowly the difficulties of this type of clinical investigation were appreciated. Moreover in 1950 erratic results were encountered both in the clinical trials and in animal experiments. It seems likely that these findings were attributable to the thermal instability of Compound I in aqueous solution in the absence of oxygen (Mitchell and Simon-Reuss, 1952). Much more consistent results have been obtained since the ampoules have been stored in a refrigerator at about 3° C.

The evidence obtained by these preliminary studies was sufficiently suggestive to justify further work, but the methods used were clearly inadequate. It became essential to employ properly designed methods for the clinical evaluation of radiosensitisers. The importance of these methods was emphasised at the time by their use in clinical evaluation of chemotherapeutic agents in tuberculosis (Medical Research Council, 1948, 1950). Investigations of this type in the study of radiosensitisers have been in progress since April 1951 ; as yet the only results available are for the treatment of inoperable carcinoma of the bronchus.

The results of the earlier studies have been reported by Mitchell (1948, 1949*a*, 1949*b*, 1950, 1951). This work has been criticised by Gellhorn and Gagliano (1950). Otte (1949) reported briefly on the combination of X-ray therapy and some synthetic vitamin K substitutes in the treatment of 300 patients with advanced malignant tumours and claimed improved palliation. Berkman (1951) obtained results very similar to those of Mitchell by the combined use of X-ray therapy and Synkavit.

*Selection of types of malignant tumour for investigation.*

It is suggested that a practical minimum requirement for usefulness of a radiosensitising chemical agent is that its employment in combination with radiotherapy should produce a mean survival time after treatment double that after radiotherapy only, for the type of malignant tumour treated. Hence taking into account the frequency distribution of the survival times, it is likely to be necessary to observe the cases treated by combined therapy for an interval of at least 6 times the mean survival after radiotherapy only. To this time must be added the time necessary to accumulate an adequate number of cases.

This approach is limited to the study of types of malignant tumour in which the results of present methods of treatment are bad and in which the time of survival is short.

Inoperable carcinoma of the bronchus is an obvious choice for investigation It is the only common type of malignant disease with a very short natural history. Despite advances, only a small proportion of all cases of carcinoma of the bronchus are curable by surgery. Untreated patients are often very miserable. The results of treatment of inoperable cases by present methods, including radical X-ray therapy, are poor. Nevertheless X-ray therapy in general has sufficient palliative value apart from prolongation of life *per se*, to justify attempts to improve this.

With X-ray therapy only, inoperable carcinoma of the bronchus appears to have a a rather uniform natural history. The results of many workers have been reviewed in a number of papers (e.g., Björk, 1947 ; Shorvon, 1947 : Ariel *et al.*, 1950). In one of the earliest studies Schinz and Zuppinger (1937) reported that for 69 cases, the mean survival time after the beginning of X-ray treatment was 4·4 months ; 16 cases (23 per cent) lived more than 6 months and 4 cases more than 1 year. Dobbie (1944) found that of 111 cases receiving palliative X-ray therapy, 26 per cent lived more than 6 months, and of 48 cases treated by radical X-ray therapy, 54 per cent lived more than 6 months after treatment. Fulton (1949) reported the following mean survival times from the onset of symptoms : for 915 untreated cases 7·6 months, for 199 cases receiving palliative X-ray therapy 9·0 months and for 190 cases treated by radical X-ray therapy, 14·7 months. The largest published series of treated cases appears to be that of Ariel *et al* (1950). The duration of life from the onset of symptoms of 723 patients who received X-ray therapy and for whom adequate data were available averaged 12·8 months. Of 816 patients receiving X-ray therapy, the average duration of life after the first X-ray treatment was 4·2 months. Of these, histological evidence was obtained in 245 patients and their average survival from the first symptom was 14·3 months and from the beginning of X-ray treatment 4·5 months. From Fig. 7 of this paper, it can be deduced that of 729 patients, only 45 survived 8 months, 20 survived 12 months, 8 survived 18 months and none survived 3 years following X-ray therapy. In this series, the best survival rate—4·7 months—was in a group of 80 patients who received a total dose as measured in air of 5000–6000 r ; higher and lower doses were associated with poorer results. The relation between survival time and tumour dose (and histology), has often been discussed, e.g., by Tenzel (1941), Adelman (1952). There appears to be a slow increase in mean survival time with increasing tumour dose except perhaps at the highest doses. Nevertheless, there is great individual variation. The survival time tends to be slightly greater in squamous carcinoma than in anaplastic carcinoma, but some cases of adenocarcinoma, especially in women, have rather longer survival. The most important factor in determining survival is the extent of spread of the disease when treated.

This evidence suggests that in representative series of cases of inoperable carcinoma of the bronchus receiving X-ray therapy, the mean survival from the beginning of treatment is about 4·5 months. Hence an investigation of a therapeutic radiosensitiser is likely to yield a definite result in this disease in the time taken to collect at least 200 cases, say 3 years in a single department, plus six times the mean survival time after radiotherapy only, which is about $2\frac{1}{4}$ years, that is at least about 5 years in all.

For any other form of malignant tumour with a substantially longer survival, direct studies of the mean survival times for radiotherapy with and without the radiosensitiser are not practicable. It would seem to be essential to devise methods of estimation of the results based on the mortality during the first few years. (cf. Boag, 1948, 1949, 1950.)

### PRELIMINARY GENERAL SURVEY

By way of approach to the problem, a general survey was undertaken of the use of Compound I mainly in conjunction with radiotherapy in the treatment of patients with various types of advanced malignant tumours except carcinoma of the bronchus. Survival studies were not practicable at this stage. The criterion of assessment of the possible value of the compound was the observation of an unexpectedly good clinical response. It is appreciated that this criterion is to some extent unsatisfactory, and not necessarily sufficiently objective. It has been applied stringently. To try to minimise its shortcomings, all the cases to which it is applied have been followed until death, or for at least $3\frac{1}{2}$ years and in assessing the results the proportion of cases showing unexpectedly good response has been compared in the case of patients treated by means of radiotherapy combined with intravenous compound with those treated by radiotherapy combined with intramuscular compound. The results of various types of treatment of patients with advanced malignant tumours classified as inoperable, Stages III and IV, and recurrent of all types except carcinoma of the bronchus using Compound I in treatments from November, 1946 to July, 1949 inclusive, and assessed up to 31 December, 1952, are summarised in Table I for the cases verified histologically. An essentially similar table has been prepared showing the rather poorer results for 56 cases not confirmed histologically, but with reasonably certain diagnosis. The cases allocated to this preliminary survey were the great majority of the patients with advanced malignant tumours in which it was considered that present methods of treatment were not likely to give satisfactorily results. When the survey was started it was not realised that the use of the compound by intravenous injection was likely to be more effective than its use by intramuscular injection. The allocation of the patients to treatments involving the two methods of administration of the compound were certainly not at random, and the possibility of bias by the use of intramuscular injection in the more seriously ill patients cannot be excluded. However, there is no reason to suppose that such bias would influence the proportion of cases showing unexpectedly good clinical response.

The results of the preliminary general survey summarised in Table I suggests that the proportion of cases showing unexpectedly good clinical response is greater with radiotherapy combined with the compound administered by intravenous injection than with radiotherapy combined with the compound administered by intramuscular injection. It is emphasised that this conclusion must not be regarded as more than suggestive.

Of the 131 cases verified histologically, 11 survived 4 years or more after treatment. Of these, 5 cases showed an unexpectedly good response. Three of these were treated by means of radical X-ray therapy combined with intravenous compound—one case of advanced carcinoma of the ovary and body of uterus with peritoneal metastases; one case of advanced reticulo-sarcoma of bones and one

TABLE I.—*General Survey.*

The results of treatment of patients with advanced malignant tumours—classified as inoperable, Stages III and IV and recurrent—of all types except carcinoma of the bronchus, treated with Compound I (Synkavit) from November, 1946, to July, 1949, and assessed to December 31, 1952. Cases verified histologically.

Number of cases showing unexpectedly good response. / Total number in group treated.

| Method of treatment. | Oral S only. | I–VS only. | Oral S previous RT. | I–MS previous RT. | I–VS previous RT. | Radical X-ray or radium therapy + I–MS. | Radical X-ray or radium therapy + I–VS. | Palliative X-ray therapy + I–MS. | Palliative X-ray therapy + I–VS. | Total number of cases at each site. |
|---|---|---|---|---|---|---|---|---|---|---|
| *Site:* | | | | | | | | | | |
| Mouth | — | — | 0/1 | 1/3 | 3/3 | 2/8 | 5/11 | 0/2 | 0/3 | 31 |
| Antrum and ethmoid | — | — | 1/1 | — | — | — | 2/4 | — | — | 5 |
| Nasopharynx | — | — | 1/1 | — | — | — | 0/1 | — | — | 2 |
| Larynx extrinsic (with 1 case intrinsic) | — | — | — | — | 0/1 | 2/4 | 1/2 | — | — | 7 |
| Post-cricoid and oesophagus | — | — | — | — | — | 1/2 | 1/2 | 0/2 | 0/1 | 7 |
| Stomach | — | 0/4 | — | — | — | — | — | — | 1/6 | 10 |
| Colon and caecum | — | 0/2 | — | — | 1/1 | — | — | 0/2 | 1/1 | 6 |
| Rectum | — | 1/1 | — | — | — | — | — | 0/2 | 0/3 | 6 |
| Cervix uteri | 0/1 | — | — | — | 1/1 | — | 2/3 | 0/1 | 0/1 | 7 |
| Body of uterus | — | — | — | — | 0/1 | — | — | — | 1/1 | 2 |
| Ovary | — | 1/1 | — | — | — | — | 2/2 | 1/5 | 0/2 | 10 |
| Breast | — | 0/1 | — | — | — | — | 0/1 | 0/4 | 0/1 | 7 |
| Skin and lip | — | — | — | — | 2/4 | 2/4 | 3/6 | — | 0/2 | 16 |
| Miscellaneous | — | 0/2 | — | — | 1/4 | 0/2 | 2/3 | 1/2 | 1/2 | 15 |
| All sites | 0/1 | 2/11 | 2/3 | 1/3 | 8/15 | 7/20 | 18/35 | 2/20 | 4/23 | 131 |

131 cases verified histologically: of these, 11 survived 4 years or more after treatment.
56 very similar cases not confirmed histologically but with reasonably certain diagnosis; of these, 2 survived more than 4 years after treatment.

187 cases in all, followed for 3½ years or more.

Summary of results:

Number of cases showing unexpectedly good response out of total number treated.

| | | | |
|---|---|---|---|
| All sites: | Radiotherapy + I–VS. | 28/77 | The difference between the two series may be regarded as suggestive if applicable, $\chi_c^2 = 3{\cdot}83$, $P = 0{\cdot}05$. |
| | Radiotherapy + I–MS. | 13/65 | |
| All sites excluding stomach and rectum: | Radiotherapy + I–VS. | 27/63 | The difference between the two series may be significant if applicable: $\chi_c^2 = 6{\cdot}19$, $P = 0{\cdot}013$. |
| | Radiotherapy + I–MS. | 13/63 | |

case of advanced neuro-fibro-lipo-sarcoma of neck. One case of advanced carcinoma of the body of the uterus was treated by means of palliative X-ray therapy combined with intravenous compound, and one case of carcinoma of the tongue recurrent after previous X-ray therapy was treated successfully by radical X-ray therapy combined with intramuscular compound.

Of the 56 cases not confirmed histologically three survived more than 4 years after treatment. Two of these showed an unexpectedly good response ; one was a woman aged 46, who had a Stage IV carcinoma of the breast with supraclavicular glands, and was treated by means of radical X-ray therapy combined with intramuscular compound, and the other was a case of a mixed parotid tumour which had failed to respond to further X-ray therapy, but appeared to be checked and rendered quiescent by intravenous compound only. An unexpected finding as shown in Table I for histologically positive cases is that 8 of 15 cases recurrent after radiotherapy showed an unespectedly good result when treated with intravenous compound only. This is the only group in which any substantial proportion of cases appeared to improve with the use of the compound alone ; this finding requires confirmation. It is emphasised that in general the compound alone has no therapeutic effect in malignant tumours.

*Preliminary Clinical Trials in Inoperable Carcinoma of the Bronchus*

The initial studies of the ancillary use of Compound I on the survival times of inoperable cases of carcinoma of the bronchus treated by X-ray therapy (Mitchell, 1948, 1951) were probably essential as a preliminary step to the design of a more satisfactory clinical trial. It is useful to summarise the end results of these studies.

An attempt was made to study the influence of the ancillary use of the compound on the results of X-ray therapy by measurement of the time of survival both after the first symptom and after the first X-ray treatment and comparison of the survival times in the *ccntrol* group of cases, which was treated by X-ray therapy only and in the *treated* group which received X-ray therapy together with the compound . The results are treated by statistical methods. It is suggested that estimation of the survival times, especially that from the beginning of X-ray therapy, is the most reliable criterion for objective assessment of the results of treatment. Special attention has been paid to the influence of the extent of the disease, age and sex, the histology, the minimum tumour dose and overall time of the X-ray therapy and the details of administration of the compound. The general principles involved in the consideration of these factors are essentially the same in these preliminary studies as in the later clinical trials and except where relevant, will be discussed in this connection. An attempt has been made to compare the effects of the compound as a radio sensitiser when administered by intramuscular and by intravenous injection.

*Selection of cases.*

The group defined as the *treated* group of cases of inoperable carcinoma of the bronchus received X-ray therapy together with the compound which was administered by intravenous and/or intramuscular injection, together with supplementary oral administration in a few cases. In the cases recorded, the first X-ray dose in the combined treatment was given in the interval from May 1, 1947

to December 31, 1949 inclusive. The results of treatment were reviewed as from December 31, 1952. This group of cases of inoperable carcinoma of the bronchus comprises all those cases referred in which the diagnosis is accepted as proven according to the criteria given below, with the exclusion of patients not likely to benefit by palliative X-ray therapy. No technically operable cases and no post-operative cases are included.

The *control* group consists of those cases of inoperable carcinoma of the bronchus selected according to the same criteria, which were treated by X-ray therapy only, using similar methods, in the same department between 1943 and May, 1947. The difficulties of this method of providing a control group are recognised and include the possibility of introduction of serious errors of sampling. The use of alternate cases as controls was attempted but was abandoned as impractical. However, it has been shown that the relevant properties of the *control* and *treated* groups apart from the length of survival after treatment are substantially the same. It is found that the results of X-ray treatment in the present *control* group are substantially identical with those obtained in two large published series. (Fulton 1949, Ariel *et al.*, 1950).

*Classification of Cases.*

In both *control* and *treated* groups the most important cases are those completely " verified histologically " by the following methods :

(*a*) bronchial biopsy ; this sub-group consists of 11 *control* cases and 26 *treated* cases.

(*b*) post-mortem examination ; this sub-group consists of 6 *control* cases and 2 *treated* cases.

(*c*) biopsy of a homolateral supra-clavicular node, in conjunction with typical bronchoscopic appearances ; in this sub-group there was one *control* case and one *treated* case.
The histological examination of the bronchial biopsy specimens was inconclusive.

(*d*) the finding of cells consistent with malignancy in the sputum, together with typical bronchoscopic appearances ; there was only one case, a *control,* in this sub-group (Table II) and in this case there were terminal metastases in the liver.

Thus of the cases described as " verified histologically," there are in all, 19 in the *control* group, which was treated by X-ray therapy only, and 29 in the *treated* group which received X-ray therapy combined with administration of the compound.

The larger proportion of cases verified histologically at post-mortem examination in the *control* group than in the *treated* group is associated with the shorter survival time and the larger proportion of cases dying in hospital.

In addition to the " histologically verified " cases, there are groups of *control* and *treated* cases in which the diagnosis is reasonably certain though less satisfactorily established. These groups are selected as follows :

(*a*) cases not verified histologically but with typical bronchoscopic appearances, together with clinical and/or radiological evidence of metastases or infiltration at any stage ; this sub-group consists of 7 *control* cases and 9 *treated* cases ;

### TABLE II.—*Clinical Therapeutic Trial of a Radiosensitiser.*

SUMMARY OF RESULTS.

Treatment of inoperable cases of carcinoma of the bronchus with randomised allocation to alternative treatments, X-ray therapy combined with intravenous Compound I (X + I–VS) and X-ray therapy combined with intramuscular Compound I (X + I–MS), which is the control group. Results assessed May 31, 1953. Cases No. 1–91 inclusive in which the first X-ray treatment was given between April 1, 1951, and December 31, 1952.

*Treatable less advanced male cases.*

Selected from these cases are those with no evidence of extra-thoracic spread or metastases in ribs at the first X-ray treatment and those in which the minimum tumour dose was not less than 1200 r. For the present purpose, cases treated surgically at any stage, and female patients are excluded. Survival is estimated in months to the nearest month from the first X-ray treatment and also from the first symptom.

| *Group.* | Number of cases surviving 8 months or more from first X-ray treatment. / Total number of cases in each group. | |
|---|---|---|
| | X + I–VS. | X + I–MS. |
| (a) Histology positive (one p.m. and sputum, others bronchial biopsy) . . . . . . | 6/8 | 4/13 |
| (b) Conclusive evidence of malignant cells in sputum and/or bronchial aspirate . . . . | 3/4* | 0/3 |
| (c) Reasonably certain diagnosis with typical bronchoscopic and radiological appearances, and clinical course . . . . . . . | 2/2 | 0/3 |
| *Pooled Groups* (a), (b) *and* (c) . . . | 11/14 | 4/19 |

* In this group is included one case surviving 4 months from the first X-ray treatment and 10 months from the first symptom which was given intra-muscular compound because of incorrect suspicion of cerebral metastases.

The difference between the proportion surviving 8 months or more from the first X-ray treatment in the two series is statistically significant; for the pooled Groups (a), (b) and (c), $\chi[_1]^2 = 10{\cdot}76$, so that $P = 0{\cdot}001$, and for the pooled Groups (a) and (b), $\chi[_1]^2 = 6{\cdot}891$, so that $P = 0{\cdot}009$.

Survival from the first symptom is a rather less sensitive and less objective test. In the pooled Groups (a), (b) and (c), the proportions surviving 12 months or more from the first symptom in the series X + I–VS and X + I–MS are respectively 11/14 and 8/19; $\chi[_1]^2 = 4{\cdot}389$, so that $P = 0{\cdot}037$, which may be regarded as significant.

| *Other cases.* | Number of cases surviving 8 months or more from first X-ray treatment. / Total number of cases in each group. | |
|---|---|---|
| | X + I–VS. | X + I–MS. |
| (d) Verified as in (a) and (b) but minimum tumour dose less than 1200 r . . . . . | 0/3 (None survived 3 months) | 0/4 (None survived 3 months) |
| (e) Male patients with extra-thoracic spread and/or metastases in ribs at first X-ray treatment . | 2/9 | 0/7 (None survived more than 4 months) |
| *Pooled Groups* (a), (b), (c), (d) *and* (e) . . | 13/26 | 4/30 |
| (f) Male patients treated surgically at some stage | 1/2 | 2/3 |
| (g) Male patients with diagnosis not verified or not bronchial carcinoma or adenoma . . . | 2/10 | 3/8 |
| (h) Female patients, all groups except bronchial adenoma . . . . . . . . | 1/5 | 1/4 |
| (i) Bronchial adenoma (both male) . . . | 0/2 | 0 |
| (j) No X-ray therapy; compound only . . | 0/1 | 0 |

For the unselected male patients with reasonably certain diagnosis in the pooled Groups (a), (b), (c), (d) and (e), the difference between the proportion surviving 8 months or more from the first X-ray treatment in the two series is statistically significant; $\chi[_1]^2 = 8{\cdot}867$, so that $P = 0{\cdot}003$. The proportions surviving 12 months or more from the first symptom in the series X + I–VS and X + I–MS are respectively 16/25† and 10/31; $\chi_c^2 = 4{\cdot}403$, so that $P = 0{\cdot}037$, which may be regarded as significant.

† The case marked * above is transferred to the series X + I–MS.

(*b*) cases with positive histology on biopsy specimens of supraclavicular nodes, cutaneous or other metastases but in which there was no bronchoscopy or the bronchoscopic appearances were not definite; this sub-group consists of 8 *control* cases and 8 *treated* cases;

(*c*) one case (*treated*) with typical bronchoscopic appearances together with metastases in the skin, confirmed histologically.

These additional 15 *control* and 18 *treated* cases are usually more advanced and are in general less comparable than the "histologically verified" cases but must be considered with them to give a complete picture. Of the combined series of "confirmed cases," there are in all 34 in the *control* group, which was treated by X-ray therapy only, and 47 in the *treated* group, which received X-ray therapy together with the compound. As anticipated the results of treatment of the "confirmed cases" are poorer than those of the "histologically verified" cases.

Other groups of cases which cannot be included in the main part of this investigation may be summarised as follows:

(i) Cases with typical radiological appearances together with metastases at some stage but not verified histologically and in which there was no bronchoscopy or the bronchoscopic appearances were not definite. In these cases the diagnosis can only be regarded as probable. In the *control* group, there are 12 cases; the mean time of survival is 8·50 months from the first symptom and 3·67 months from the first X-ray treatment. In the *treated* group, there are 13 cases; the mean times of survival are 12·15 months from the first symptom and 6·31 months from the first X-ray treatment.

(ii) Cases rejected as unsuitable for palliative X-ray therapy. During the *control* period, there were 18 of these cases, of which 10 were histologically verified and during the period corresponding to the *treated* group, there were 7 cases of which only one was verified histologically.

The enumeration of rejected cases is unsatisfactory because of selection by the physicians referring these advanced cases.

Hence the total number of cases with a reasonably certain or probable diagnosis of inoperable carcinoma of the bronchus is 131. The *control* group is drawn from a total of 64 cases, and the *treated* group from a total of 67 cases.

*Histology.*

Of the 19 verified *control* cases, there are 11 cases of stratified carcinoma, of which 7 are squamous and 4 non-squamous, 3 cases of adenocarcinoma, 4 of ana plastic carcinoma and one unclassified. Of the 7 cases of squamous carcinoma, two showed keratinisation.

Of the 29 verified *treated* cases, there are 17 cases of stratified carcinoma, of which 10 are squamous and 7 non-squamous, 3 cases of adenocarcinoma, 8 of anaplastic carcinoma and one an unusual poorly differentiated lesion.

Of the 10 cases of squamous carcinoma three cases show definite keratinisation; in two further cases there is a suggestion of keratinisation. It is interesting to note that all these cases responded well to treatment.

### RESULTS.

The *control* group was treated by X-ray therapy only, from 1943 to May, 1947. The *treated* group was treated by X-ray therapy combined with tetrasodium 2-methyl-1 : 4-naphthohydroquinone diphosphate (Compound I, Synkavit) administered by intravenous and/or intramuscular injection from May, 1947 to December, 1949 inclusive.

*Cases verified histologically.*

Cases were verified histologically mainly by bronchial biopsy and/or post-mortem examination.

*Control* group : X-ray therapy only.

19 *cases* : mean survival 3·89 months from the first X-ray treatment and 11·00 months from the first symptom.

Only 1 case, who survived 15 months, survived more than 8 months from the first X-ray treatment.

Three cases survived more than 15 months from the first symptom.

*Treated* group : X-ray therapy combined with the compound.

29 *cases* : mean survival 9·38 months from the first X-ray treatment and 17·55 months from the first symptom.

Of these, 15 cases survived more than 18 months and 7 cases more than 15 months from the first treatment ; 15 cases also survived more than 15 months from the first symptom.

The survival time from the first X-ray treatment is significantly greater in the *treated* group (mean survival 9·38 months) than in the *control* group (mean survival 3·89 months)—($n = 46$ Student's $t = 3{\cdot}519$, $P = 0{\cdot}001$ ; using the logarithms of the survival times, $t = 4{\cdot}046$). Even if the cases which survived only one month from the first X-ray treatment are excluded, to meet the criticism of a possible difference in selection of the cases, the difference in survival after the first treatment between the *treated* group (mean survival 9·68 months) and the *control* group (mean survival 5·23 months) is still significant ($n = 39$, $t = 2{\cdot}457$, $P$ is slightly less than 0·02). Considering the histological classification, the only type providing adequate numbers is the stratified carcinoma. The proportion of cases of stratified carcinoma is almost identical in the *control* (11/19) and *treated* (17/29) groups. Of the cases of stratified carcinoma surviving more than 8 months from the first X-ray treatment, there are 1 out of 11 cases in the control group and 11 out of 17 cases in the *treated* group ; the survival must be regarded as significantly greater in the *treated* than in the *control* group ; ($\chi_c^2 = 6{\cdot}32$, $P = 0{\cdot}012$).

It is relevant to note that the frequency distribution of survival times after the first X-ray treatment is positively J-shaped for the *control* group and roughly symmetrical, probably approximating to lognormal for the *treated* group. There are significantly fewer short survivals in the *treated* group than in the *control* group, e.g., of cases surviving 3 months or less after the first X-ray treatment, there were 5/29 in the former and 12/19 in the latter ; $\chi_c^2 = 8{\cdot}67$, $P = 0{\cdot}003$. It is suggested that this change in frequency distribution provides further evidence for the reality of the radiosensitisation.

The survival time from the first symptom is significantly greater in the *treated* group (mean survival 17·55 months) than in the *control* group (mean survival 11·00 months)—($n = 46$, $t = 2{\cdot}624$, $P = 0{\cdot}012$). There is no significant difference between the time interval from the first symptom to the first treatment in the *control* group (mean interval 7·11 months) and in the *treated* group (mean interval 8·17 months). It is to be noted that the *treated* group includes one case, who had a history of 45 months' duration before treatment and survived 3 months from the first X-ray treatment.

Within the *treated* group, 20 cases were treated by X-ray therapy combined with the compound administered by intravenous injection or by both intramuscular and intravenous injection. In this *treated* sub-group of 20 cases, the mean survival time is 11·05 months and is significantly greater than the mean survival time of 3·89 months in the *control* group of 19 cases ($n = 37, t = 4{\cdot}345, P < 0{\cdot}001$).

There is suggestive evidence that X-ray therapy combined with *intravenous* compound is associated with longer survival after the first X-ray treatment (18 cases, mean survival 11·17 months) than is X-ray therapy combined with intramuscular compound (11 cases, mean survival 6·45 months)—($n = 27$, $t = 2{\cdot}106$, $P = 0{\cdot}04$).

*All confirmed cases.*

*Control* group : X-ray therapy only.

34 *cases* : mean survival 3·82 months from the first X-ray treatment and 10·00 months from the first symptom.

Only 2 cases, which survived 12 and 15 months respectively, survived more than 8 months from the first X-ray treatment and 4 cases survived more than 15 months from the first symptom.

*Treated* group : X-ray therapy combined with the compound.

47 *cases* : mean survival 8·72 months from the first X-ray treatment and 15·45 months from the first symptom.

Of these, 18 cases survived more than 8 months and 8 cases more than 15 months from the first X-ray treatment ; 16 cases survived more than 15 months from the first symptom.

The survival time from the first X-ray treatment is significantly greater in the *treated* group (mean survival 8·73 months) than in the *control* group (mean survival 3·82 months) — ($n = 79$, $t = 3{\cdot}630$, $P < 0{\cdot}001$ ; using the logarithms of the survival times, $t = 3{\cdot}967$).

The survival time from the first symptom is also to be regarded as significantly greater in the *treated* group (mean survival 15·45 months) than in the *control* group (mean survival 10·00 months)—($n = 79$, $t = 2{\cdot}678$, $P < 0{\cdot}01$).

It is interesting to consider the proportion of cases surviving more than 15 months from the first X-ray treatment out of the total number of cases with reasonably certain or probable diagnosis and including those cases rejected as unsuitable for X-ray therapy during the periods of observation. In these total groups, there are 64 *control* cases (including 8 rejected) of whom none survived more than 15 months from the first X-ray treatment and 67 *treated* cases (including 7 rejected) of whom 9 survived more than 15 months. This difference between the *control* and *treated* cases is significant ($\chi_c^2 = 7{\cdot}25$, $P = 0{\cdot}008$ and excluding the cases rejected, $\chi_c^2 = 5{\cdot}73$, $P = 0{\cdot}017$).

In the group of confirmed cases, 6 *control* cases and 6 *treated* cases received radical X-ray therapy. For these *control* cases, the mean survival time is 14·5 months from the first symptom and 6·3 months from the first X-ray treatment. For the *treated* cases the mean survival time is greater than 22·5 months from the first symptom and greater than 13·2 months from the first treatment. However, the number of these cases is too small for the observed difference to be taken as a real one with any certainty. It is interesting to note that the mean survival from the first symptom in Fulton's (1949) series of 190 cases treated by radical X-ray therapy was 14·7 months ; the closeness of the aggreement is fortuitous.

This preliminary trial suggests that in the treatment of inoperable carcinoma of the bronchus the ancillary use of Compound I produces a small but useful increase in the length of survival. This finding is in accordance with the clinical impressions and the small improvement in length of survival appears to be associated with useful life.

However, this study is not entirely satisfactory in that the *control* group treated by X-ray therapy only and the *treated* group which received X-ray therapy combined with the compound, were treated consecutively and not within the same period of time.

### *Design of a Clinical Therapeutic Trial of a Radiosensitiser.*

The design of a clinical trial to evaluate any proposed method of treatment of cancer appears to be one of the most important problems in medicine at the present time. The basic principles involved are well understood as a result of the classical statistical studies of Fisher (1925, 1935) on the design of experiments, and on the work of Hill (1952) on the design of clinical trials. In general, in considering therapeutic trials in the cancer field, the design must be capable of giving a completely convincing statistically significant quantitative result as efficiently as possible with a minimum number of patients and must be acceptable and above reproach from the moral and ethical points of view. The most important single feature of the design is random allocation of the patients to two or more alternative forms of treatment the value of which is being compared. For assessment of the result of treatment, it is necessary to select relevant criteria capable of objective measurement such as the survival time suitably specified. At the same time, it is absolutely essential to provide for each individual patient treatment which is the best possible according to present knowledge. Accordingly, it may be, and probably often will be, necessary to plan to depart from what may be termed the theoretically ideal form of experiment with deliberate sacrifice of some information.

In the present example of the attempted evaluation of tetra-sodium 2-methyl-1 : 4-naphthohydroquinone diphosphate (Compound I, Synkavit) as a radiosensitiser in the radio therapy of inoperable carcinoma of the bronchus, cases were allocated at random to one of two alternative forms of treatment, X-ray therapy combined with Compound I administered by *intravenous* injection (X + I–VS) and X-ray therapy combined with Compound I administered by *intramuscular* injection (X + I–MS). The latter is regarded as the control group. The preliminary clinical studies and animal experiments suggested that the results of X-ray therapy combined with *intramuscular* compound (X + I–MS) were slightly better than those of X-ray therapy only, and that with comparable doses of compound it was likely that X + I–VS would produce better results than X + I–MS. The use of X + I–MS as the control group instead of, e.g., X-ray therapy combined with distilled water, almost certainly scarifices a small amount of information but was regarded as essential in order to give to each individual patient the best available proven form of treatment. This type of design of a clinical therapeutic trial must be justified *a posteriori*. It is necessary to examine the possible importance of all the various factors which may be relevant including age, sex, extent of the disease at treatment, histology and/or cytology, the minimum tumour dose and overall time of radiotherapy, the total dose and overall time of administration of the compound and in particular the certainty of the diagnosis. Tests of heterogeneity must be made. It does not seem possible to estimate in advance the number of patients required. This will depend on the differences observed between the groups and subgroups. The results are likely to be of sufficient importance to necessitate the use of stringent criteria of significance.

The randomisation was made on the basis of a provisional diagnosis of inoperable carcinoma of the bronchus, made when the patient was first examined by the staff of the Department. Some of the cases were referred with histological evidence from bronchial biopsy. In other cases, there had been no previous investigation and the provisional diagnosis was based on clinical evidence only.

All cases were investigated critically, as necessary, and every effort was made to obtain histological and/or cytological verification of the diagnosis. Almost all the sections have been reviewed by Dr. A. M. Barrett, of the Department of Pathology of the University of Cambridge. The samples of sputum have been examined mainly by Dr. J. H Dean. I am indebted to Dr. D. B. Cruickshank, of the Sims Woodhead Memorial Laboratory, Papworth, for most of the reports on specimens of bronchial aspirate. Particular attention has been paid to the establishment of the diagnosis of carcinoma of the bronchus and the identification and exclusion of cases of bronchial adenoma and related tumours of low grade malignancy apparently arising in bronchial glands. The histological classification of carcinoma of the bronchus is that employed by Dr. A. M. Barrett and may be summarised as follows:

(1) Stratified carcinoma, either squamous or non-squamous, the degree of keratinisation or differentiation being noted, (2) adenocarcinoma, (3) anaplastic carcinoma, including "oat-celled" carcinoma, and (4) others, including "alveolar" cell carcinoma and carcinoma not classifiable. The histological classification of carcinoma of the bronchus is often difficult because of the existence of intermediate types and is somewhat arbitrary in some cases.

On the clinical side, a careful and full medical examination is made, including usually indirect laryngoscopy. It has been considered important to determine the extent of spread of the disease with special reference to the early detection of metastases in bones. The occasional cases originally considered inoperable and in which it has been found possible subsequently to carry out some type of surgery have been considered separately in the assessment of the results. The fate of every case included in the trial must be explicitly stated.

To obtain randomisation, the serial number of the patient in the special register of cases of inoperable carcinoma of the bronchus was taken and the corresponding number read off in a table of random numbers; Table xxxiii (vi) in Fisher and Yates (1948, p. 109) was used. For random numbers ending with an even number or zero, the patient was allocated to receive X-ray therapy combined with the compound administered by intravenous injection (X + I–VS); for odd random numbers, the patient was allocated to receive X-ray therapy combined with intramuscular compound (X + I–MS). If there are medical contraindications to intravenous administration of the compound including cerebral metastases and threatened laryngeal obstruction, where the possible risk of increased oedema might be dangerous, severe myocardial degeneration and impossible veins, and the random number corresponds to X + I–VS, the compound is given by intramuscular injection, but the case is still classified as X + I–VS, with a suitable note. Similarly it may occasionally be desirable to administer the compound by intravenous injection to an individual patient in the group X + I–MS. This type of procedure avoids bias in that it leads to underestimation of the difference between the two groups. If any case is retreated, the compound is administered by the route previously used.

For the purpose of this trial, the upper level of dosage of the compound administered by intramuscular injection is kept at 100 mg. daily, while for intravenous administration the dose is increased to as high a level as is tolerated. For both I–M and I–V, the dosage is started with 25 mg. and increased daily in steps of 25 mg. If any undesirable reaction such as excessive coughing, nausea, faintness or discomfort or pain in the chest is observed after intravenous injection, the dose is reduced to a level at which there is no such reaction and then cautiously increased again on the following days, sometimes by steps of 10 mg. For most patients, for intravenous injections the best maximum daily dose appears to be 100 mg.; many will tolerate 150 mg. and a few will tolerate 250 mg. It is often necesssary at the higher dosage levels to reduce the daily dose after a time.

With regard to timing, in the series of cases the injections have been given as closely as possible to 30 minutes before starting the individual X-ray treatment. In many cases it was only possible to start the injections on the day of the first X-ray treatment, but whenever practicable, thei njections have been commenced 3–5 days before starting the X-ray therapy in order to reach the higher dosage levels. In general, the injections are continued daily without interruption during the course of X-ray therapy and are stopped with the last X-ray treatment. The compound is never administered orally in this trial.

In this trial, every endeavour has been made to employ the normal methods of X-ray therapy and to avoid any modification of the techniques as a result of the use of the compound. The X-ray therapy has been of conventional type using filtered 220 kVp X-radiation of H.V.L. 1·5 mm. copper, with radiographic checking of the thoracic field positions in most cases. In a large proportion of the cases only palliative X-ray therapy was considered justifiable; the commonest technique used was with two fields, anterior and posterior thoracic, usually 15 × 15 cm. or 12·5 cm. diameter, with central minimum tumour dose 1500–2000 r given in 7 treatments in overall time 8 days. Radical X-ray therapy has been limited usually to patients in good general condition with a lesion considered to be localised though inoperable. For radical X-ray therapy, and also for palliative X-ray therapy in some cases, a four-field or six-field technique with beam direction has been used; the field size varied between 8 × 6 cm. and 15 × 10 cm. but that used most often was 15 × 7 cm. The treatment has been regarded as radical when the minimum tumour dose is 3000 r or greater in overall time 42 days or less. It is to be noted that for most of the cases with overall time about 39 days, the treatment was of "spaced" type, with 5 consecutive daily treatments, 9 days interval, then 5 consecutive daily treatments, 16 days interval and finally 5 more treatments. A treatment with M.T.D. 2400 r in O.T. 18 days has been classed as palliative and M.T.D. 5500 r in O.T. 58 days as radical.

With regard to general medical treatment, pyridoxine has not been used for radiation sickness, and the nitrogen mustards have not been used for attempted palliation of symptoms.

### RESULTS TO DATE.

The results of this clinical trial as assessed on 31 May, 1953, are summarised in Table II. This deals with cases No. 1–91 inclusive in which the first X-ray treatment was given between 1 April, 1951 and 31 December, 1952. Some cases are still surviving so that additional evidence concerning the diagnosis may be forthcoming in a few of these and the estimates of survival time are necessarily incomplete. It is reasonably certain that any such new evidence will increase the differences observed between the two series.

The total numbers of cases are 46 in the X + I–VS series, of which one received compound only with no X-ray therapy and 45 in the X + I–MS series. The fate of every case is accounted for in the Groups (a) to (j) of Table II. The small groups of male patients treated surgically at some stage (f) and female patients (h) are too heterogeneous and too small to be of value in investigation. Accurate diagnosis is of extreme importance in any studies of the treatment of carcinoma of the bronchus and appears to present greater difficulties than are commonly appreciated. The interpretation of bronchial biopsies is often difficult and review of the sections has led to rejection of the diagnosis of bronchial carcinoma in three cases (No. 45, 63, 68). Positive histology on metastases in supraclavicular nodes, skin or bone is not necessarily evidence that the primary tumour is in a bronchus. Stringent criteria have been used in the cytological evaluation of malignant cells in sputum and bronchial aspirate. It is interesting that in Group (g) in which the diagnosis of bronchial carcinoma was unverified or probably incorrect, the results included some which at first consideration appeared to be among the most satisfactory.

The evidence to date presented in Table II shows that both for the selected treatable male patients with less advanced inoperable carcinoma of the bronchus in Groups (a), (b) and (c), and for all the male patients, not treated surgically and with reasonably certain diagnosis in the Groups (a), (b), (c), (d) and (e), a substantially larger proportion survive 8 months or more from the first X-ray treatment when this is combined with intravenous Compound I than when combined with intramuscular compound. The proportions are 11/14 for X + IV–S as compared with 4/19 for X + I–MS for pooled Groups (a), (b) and (c), and 13/26 for X + I–VS as compared with 4/30 for X + I–MS for the pooled Groups (a) to (e). Survival statistics are as yet incomplete but in the pooled Groups (a), (b) and (c)—with transfer of the case marked * to the X + I–MS series —the estimated survival time from the first X-ray treatment is significantly larger in the X + I–VS series (mean survival 10·8 months) than in the X + I–MS series (mean survival 5·9 months)—($n = 31, t = 2{\cdot}701$, so that $P = 0{\cdot}011$). As the number of cases increases, the significance of the various differences between the two series increases.

Survival from the first symptom is a less sensitive and less objective test. In some of the groups, the differences between the times of survival from the first symptom in the two series are suggestive but not really convincing. For the pooled Groups (a), (b), (c), (d) and (e), if the case marked * is transferred to the series X + I–MS, the proportions surviving 12 months or more from the first recorded symptom in the series X + I–VS and X + I–MS are respectively 16/25 and 10/31; with Yates's correction for continuity $\chi_c^2 = 4{\cdot}403$ so that $P = 0{\cdot}037$; thus the difference between these two groups can be regarded as significant.

It is necessary to consider the composition of the various groups in the two series in Table I. Histological classification is possible for all cases in Groups (a) and (b). There were no cases of adenocarcinoma. In the X + I–VS series, there were 9 cases of stratified carcinoma—7 squamous including one mixed form—and 7/9 including one anaplastic carcinoma survived 8 months or more from the first X-ray treatment. In the X + I–MS series, there were 14 cases of stratified carcinoma of which 11 were squamous and of which only 4, 3 squamous and one non-squamous, survived 8 months or more from the first X-ray treatment. The difference in survival in these small groups in the two series is suggestive.

The methods of X-ray therapy and administration of the compound are not significantly different in the two series and fortunately such differences as are found might be considered as tending in all instances to reduce the observed differences in survival between the two series. Considering the Groups (a), (b) and (c), for the 14 patients in the X + I–VS series, only one received radical treatment, and this case survived 8 months. The mean M.T.D. was 1,806 r delivered in mean O.T. 11·4 days; and the mean amount of compound used was 757 mg. administered in mean O.T. 12·4 days; for the 19 patients in the X + I–MS series, 4 received radical treatment and survived respectively 4, 6, 7 and 6 months, the mean M.T.D. was 2,065 r delivered in mean O.T. 18·5 days and the mean amount of compound used was 950 mg. administered in mean O.T. 20 days. The age at treatment in the X + I–VS series had a mean of 58·1 years with extremes of 44 and 72 years, and in the X + I–MS series, a mean of 52·8 years with extremes of 40 and 68 years.

None of these differences between the two series is statistically significant. For 31 degrees of freedom, for the differences for mean M.T.D., $t = 0{\cdot}924$ and $P = 0{\cdot}36$; for mean overall time of X-ray therapy $t = 1{\cdot}486$, and $P = 0{\cdot}15$, for mean amount of Compound $t = 1{\cdot}01$ and $P = 0{\cdot}32$; for mean overall time of administration of Compound $t = 1{\cdot}602$ and $P = 0{\cdot}11$; and for mean age at the first treatment $t = 1{\cdot}621$ and $P = 0{\cdot}11$.

Accordingly, it would appear to be justifiable to conclude that the results obtained so far in this clinical trial indicate that intravenous administration of Compound I (Synkavit) has a small but useful effect as a clinical radiosensitiser in the radiotherapy of inoperable cases of carcinoma of the bronchus. However, further evidence is desirable.

### SUMMARY.

An attempt has been made to assess the clinical value of tetra-sodium 2-methyl-1 : 4-naphthohydroquinone diphosphate (Synkavit) as a radiosensitiser in the radiotherapy of malignant tumours.

A preliminary general survey of the use of the compound in the treatment of patients with various types of malignant tumour other than carcinoma of the bronchus with follow-up for at least three and a half years is summarised. The results suggest that the proportion of cases showing unexpectedly good clinical response is greater with radiotherapy combined with the compound administered by intravenous injection than with radiotherapy combined with the compound administered by intramuscular injection.

Preliminary clinical studies of the influence of the ancillary use of the compound on the survival times of inoperable cases of carcinoma of the bronchus treated by X-ray therapy are discussed in some detail and the end results summarised.

The design of a clinical therapeutic trial of a radiosensitiser is discussed, with special reference to the evaluation of tetra-sodium 2-methyl-1 : 4-naphthohydroquinone diphosphate as a radiosensitiser in the radiotherapy of inoperable cases of carcinoma of the bronchus. The results of this clinical trial are summarised to date.

It is concluded that intravenous administration of this compound (Synkavit) has a small but useful effect as a clinical radiosensitiser.

In this work I have been helped by many colleagues at Addenbrooke's Hospital. In particular I wish to thank Dr. A. M. Barrett and Dr. W. Paton Philip, and Miss E. J. Porter, Sister-in-Charge of the Radiotherapeutic Centre and her staff.

I wish to thank Dr. F. Wrigley and Dr. A. L. Morrison, of Roche Products Limited, Wewyn Garden City, for supplies of Synkavit.

### REFERENCES.

ADELMAN, B. P.—(1952) *Radiology*, **59**, 390.

ARIEL, I. M., AVERY, E. E., KANTER, L., HEAD, J. R., AND LANGSTON, H. T.—(1950) *Cancer*, **3**, 229.

BERKMAN, A. TEVFIK—(1951) Türkiye Tip Encümeni Arşivi (Istanbul), **2**, 1 (and private communication).

BOAG, J. W.—(1948) *Brit. J. Radiol.* **31**, 128, 189.—(1949) *J. Roy. statist. Soc.*, B, **11**, 15.—(1950) *Spec. Rep. Ser., med. Res. Coun. Lond.*, No. 267 (by C. A. P. Wood and J. W. Boag). Appendix A, p. 138.

BJÖRK, V. O.—(1947) *Acta Chir. scand.*, **95**, Suppl. 123, chapter 6, p. 77.

DOBBIE, J. L.—(1944) *Brit. J. Radiol.*, **17**, 107.

FISHER, R. A.—(1925) 'Statistical Methods for Research Workers.' Edinburgh (Oliver and Boyd, Ltd.).—(1935) 'The Design of Experiments.' Edinburgh (Oliver and Boyd, Ltd.).

*Idem* AND YATES, F.—(1948) 'Statistical Tables for Biological, Agricultural and Medical Research.' Third edition. Edinburgh (Oliver and Boyd, Ltd.).

FULTON, J. S.—(1949) *Proc. Roy. Soc. Med.*, **42**, 775.

GELLHORN, A., AND GAGLIANO, T.—(1950) *Brit. J. Cancer*, **4**, 103.

HILL, A. B.—(1952) 'Principles of Medical Statistics.' London (Lancet, Ltd.). Fifth edn., p. 5, 233.

MEDICAL RESEARCH COUNCIL—(1948) *Brit. med. J.*, *ii*, 769.—(1950) *Ibid.*, *ii*, 1073.

MITCHELL, J. S.—(1948) *Brit. J. Cancer*, **2**, 351.—(1949*a*) *Experientia*, **5**, 293.—(1949*b*) *Ann. Rep. Brit. Emp. Cancer Campgn.*, **27**, 214.—(1950) *Ibid.*, **28**, 213.—(1951) *Ibid.*, **29**, 190.

*Idem* AND SIMON-REUSS, I.—(1952) *Brit. J. Cancer*, **6**, 305.

OTTE, J. W.—(1949) First International Congress of Biochemistry, Cambridge. Discussion.

SCHINZ, H. R., AND ZUPPINGER, A.—(1937) 'Siebzehn Jahre Strahlentherapie der Krebse.' Leipzig (Thieme), p. 255.

SHORVON, L. M.—(1947) *Brit. J. Radiol.*, **20**, 443.

TENZEL, W. V.—(1941) *J. Amer. med. Ass.*, **117**, 1778.